# Response to Commentary On: Encoding Genes of Metallo‐β‐Lactamases (*IMP*, *NDM*, and *VIM*) in *Klebsiella pneumoniae* in Iran: A Systematic Review and Meta‐Analysis

**DOI:** 10.1002/hsr2.71357

**Published:** 2025-10-07

**Authors:** Hamid Sadeghi, Saeideh Gholamzadeh khoei

**Affiliations:** ^1^ Medical Microbiology Research Center Qazvin University of Medical Sciences Qazvin Iran; ^2^ Student Research Committee Qazvin University of Medical Sciences Qazvin Iran

## Data Availability Statement

The data that support the findings of this study are available from the corresponding author upon reasonable request. The data that supports the findings of this study are available upon request to the authors.

## Transparency Statement

The authors, Hamid Sadeghi and Saeideh Gholamzadeh Khoei, confirm that the issues addressed in this response are presented transparently. All explanations and clarifications are provided honestly and without selective reporting.

Dear Editor,

We would like to thank the authors of the letter entitled *“*Encoding Genes of Metallo‐β‐lactamases (*IMP*, *NDM*, and *VIM*) in *Klebsiella pneumoniae* in Iran: A Systematic Review and Meta‐analysis*”* for their thoughtful and constructive feedback on our recent publication [1]. Their comments raise important methodological and contextual considerations that deepen the ongoing discourse on antimicrobial resistance (AMR) surveillance, particularly in resource limited settings. With regard to the observed high heterogeneity (*I*² > 70%) across the three genes, we agree this is a common feature in meta‐analysis involving diverse geographic areas, long study periods, and multiple outcomes. As noted in the literature, elevated *I*² values are typical in prevalence studies and do not necessarily reflect problematic heterogeneity [2]. To address this, we applied a random‐effects model throughout our analyses, in line with methodological best practices [3]. To assess geographic variation, subgroup analyses by city were performed separately for each gene (*NDM*, *VIM*, and *IMP*), resulting in three distinct subgroups. This approach allowed meaningful stratification while maintaining statistical robustness. Additional subgrouping was avoided to prevent data fragmentation, reduced power, and misleading interpretations, consistent with prior research warnings on excessive stratification in meta‐analysis [4]. All included studies employed molecular detection methods based on PCR, as specified in our inclusion criteria and detailed in Table 1 [1]; therefore, subgroup analysis by detection method were not applicable. All included studies were selected based on molecular detection via PCR, as outlined in our criteria. While minor differences in PCR protocols (primers or amplification conditions) may exist, the diagnostic platform remained consistent, minimizing major methodological heterogeneity. This variation was acknowledged in the limitations section.

**Table 1:** Main characteristics of the included studies reporting the prevalence of MBL Genes in *Klebsiella Penumoniae*.

N	Study name	Publication year	Source of sample	Sample size	Genotype methods	MBL genes			Iranian province
						IMP	VIM	NDM	
1	H Zeighami	2014	Clinical sample	149	PCR Amplification	12	5	NI	Zanjan
2	H Alizadeh	2021	Clinical sample	240	PCR Amplification	2	11	2	Isfahan
3	S Armin	2018	Clinical sample	145	PCR Amplification	ND	ND	4	Tabriz/Mashhad
4	S Armin	2021	Clinical sample	230	PCR Amplification	1	ND	29	Ten Provinces
5	E Abbasi	2023	Clinical sample	121	PCR Amplification	ND	ND	36	Arak
6	N Bahmani	2019	Clinical sample	114	PCR Amplification	1	4	NI	Kurdistan
7	A Bahramian	2019	Clinical sample	120	PCR Amplification	ND	ND	3	Tehran
8	N Darabi	2019	Clinical sample	182	PCR Amplification	3	22	7	Urmia
9	S Davoudabadi	2023	Clinical sample	52	PCR Amplification	ND	ND	6	Tehran
10	H Fazeli	2015	Clinical sample	112	PCR Amplification	ND	ND	6	Isfahan
11	F Firoozeh	2017	Clinical sample	181	PCR Amplification	NI	NI	20	Kashan
12	A Hashemi	2014	Clinical sample	83	PCR Amplification	NI	NI	ND	Tehran
13	Z Hosseinzadeh	2017	Clinical sample	211	PCR Amplification	ND	NI	27	Shiraz
14	S Jamali	2020	Clinical sample	68	PCR Amplification	NI	NI	8	Guilan
15	S Kadivarian	2023	Clinical sample	62	PCR Amplification	1	ND	NI	Kermanshah
16	H Kazemian	2019	Clinical sample	90	PCR Amplification	1	30	ND	Tehran/Ilam
17	S Kiaei	2018	Clinical sample	175	PCR Amplification	ND	ND	37	Kerman
18	B Latifi	2020	Clinical sample	151	PCR Amplification	ND	ND	11	Bushehr
19	M Moghadampour	2018	Clinical sample	80	PCR Amplification	ND	ND	8	Isfahan
20	S Nobari	2014	Clinical sample	180	PCR Amplification	ND	5	3	Tehran
21	R Rajabnia	2015	Clinical sample	50	PCR Amplification	NI	15	NI	Babol
22	F Shahcheraghi	2012	Clinical sample	45	PCR Amplification	NI	NI	1	Tehran
23	S Shoja	2022	Clinical sample	400	PCR Amplification	ND	ND	30	Bandar Abbas
24	S Shoja	2017	Clinical sample	170	PCR Amplification	ND	ND	4	Bandar Abbas
25	F Riyahi Zaniani	2022	Clinical sample	32	PCR Amplification	6	ND	9	Dezful

Abbreviations: ND, not detected; NI, not investigated.

Meta‐regression to examine temporal trends was conducted only for the most prevalent gene, *NDM*, revealing a significant association between prevalence and publication year (*p* = 0.009), indicating a temporal effect. As noted in the limitations section of our original article, sources of variability such as diagnostic protocols and regional sample distribution were acknowledged, and readers were advised to interpret results with caution. Overall, our methodological choices balance comprehensive analysis with the limitations of available data to ensure clarity and robustness.

**Figure 1:**
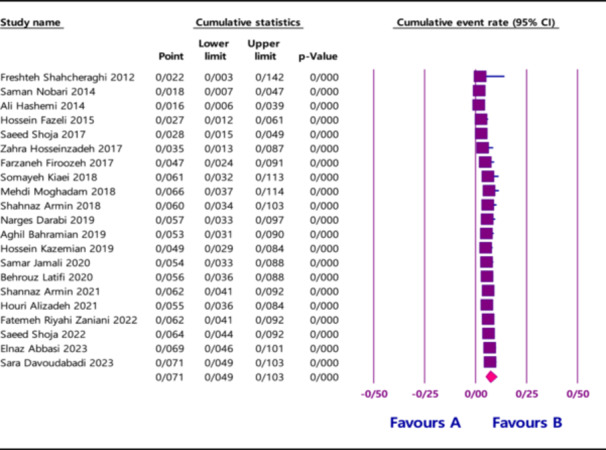
Forest plots for the prevalence of *NDM* during the time.

In response to the concern regarding omission of temporal trend analysis over the 14‐year study period, we clarify that such an analysis was indeed performed. Specifically, changes in *NDM* prevalence over time were assessed visually, as shown in Figure 1 [1] and described in Section 3.5 (Time Series Analysis) [1]. Our findings indicate fluctuations in *NDM* prevalence, including a slight decrease from 2012 to 2014, a significant increase between 2015 and 2018, a minor decline from 2018 to 2019, and a subsequent rise from 2019 to 2023. These results align with global concerns over rising *NDM*‐type carbapenemases and provide valuable epidemiological insights for resource allocation and policy planning.

We recognize the clinical importance of phenotypic data such as Minimum inhibitory concentration (MIC) values and genotype‐phenotype correlations. However, a significant methodological challenge limits the comparability and integration of phenotypic data (Disk diffusion and MIC) across studies spanning a long timeframe [5]. Organizations such as Clinical and Laboratory Standards Institute (CLSI) and European Committee on Antimicrobial Susceptibility Testing (EUCAST) regularly revise and update MIC breakpoints defining bacterial susceptibility categories [6, 7]. For example, an MIC value considered “intermediate” in 2012 might be classified as “resistant” in 2022 due to these updates. Therefore, when combining MIC data from studies conducted between 2010 and 2024, it is often unclear which breakpoint version was applied, making direct comparison and meta‐analytic synthesis potentially misleading or invalid. Given these periodic changes affecting phenotypic interpretations [6], and considering that genes are independent of breakpoint revisions and provide greater stability [8], we focused on gene prevalence rather than phenotypic resistance patterns to maintain methodological consistency and robustness.

We acknowledge the point regarding the exclusion of gray literature and hospital datasets, which may offer valuable data, especially from under‐resourced settings with significant clinical challenges posed by Carbapenem‐Resistant *Klebsiella pneumoniae* (CRKP). However, systematic reviews suggest that excluding non‐English and gray literature generally has minimal impact on conclusions and can be practical for rapid reviews [9, 10]. Still, we agree that in the context of AMR in low resource regions, such exclusion may risk underrepresentation or overestimation of prevalence highlighting the need for improved data collection and inclusion in future studies.

Our decision to exclude gray literature was based on concerns over data quality, as many such sources (e.g., non‐indexed local journals) lack standardized peer review and methodological transparency. Including them could introduce bias, so to ensure scientific integrity, we limited our analysis to peer‐reviewed studies indexed in recognized databases. Nevertheless, we acknowledge this approach may omit valuable data, as indicated by potential publication bias in funnel plot asymmetry and Egger's and Begg's tests.

We also recognize the importance of integrating molecular prevalence data with clinical outcomes and antibiotic usage to inform antimicrobial stewardship, particularly in resource‐limited settings like many Iranian regions. Unfortunately, existing literature lacked sufficient detail to support such analysis.

In conclusion, we appreciate the Authors' recognition of the importance of our study in shedding light on the molecular epidemiology of MBL‐encoding genes in Iran. We concur that future research would benefit from enhanced analytical stratification, temporal modeling, and integration with clinical data to translate molecular surveillance into actionable national AMR control strategies.

## Author Contributions

**Hamid Sadeghi:** writing – review and editing. **Saeideh Gholamzadeh khoei:** writing – review and editing, conceptualization.

## Ethics Statement

All authors have read and approved the final version of this manuscript. The corresponding author, Saeideh Gholamzadeh Khoei, had full responsibility for the integrity of the content and affirms the accuracy of this response.

## Conflicts of Interest

The authors declare no conflicts of interest.
